# Circ-0036602 Acts As a Sponge of MiR-34a-5p and MiR-431-5p to Promote Cervical Cancer Proliferation and Invasion

**DOI:** 10.7150/jgen.62458

**Published:** 2022-01-11

**Authors:** Xia Ye, Biqing Zhu, Jingjing Han, Jian Huang, Yaqin Wu

**Affiliations:** 1 Department of Radiation Oncology, The First People's Hospital of Nantong, The Second Affiliated Hospital of Nantong University, Nantong 226001, China.; 2 Department of Radiation Oncology, Jiangsu Cancer Hospital, Jiangsu Institute of Cancer Research, The Affiliated Cancer Hospital of Nanjing Medical University, Nanjing 210009, China.

**Keywords:** Cervical cancer, circ0036602, circRNA, HPV, invasion and metastasis

## Abstract

**Background**: Cervical cancer (CC) is one of the most common female malignancies worldwide. An increasing body of evidence suggests that circular RNAs (circRNAs) participate in the pathogenesis of various cancers, including CC. However, the expression profile and underlying molecular mechanisms remain largely unknown.

**Methods**: In this study, high throughput sequencing was applied to identify circRNA in HPV-16 positive CC tissues. Quantitative real-time PCR (qRT-PCR) was performed to validate the expression in CC tissues and cell lines. RNase R treatment, gel-electrophoresis, and RNA fluorescent *in situ* hybridization (FISH) were used to characterize the circRNAs. Subsequently, the Cell Counting Kit-8 assay (CCK8), transwell and wound healing assays were performed to assess circRNA function. Meanwhile, dual-luciferase reporter and western blot were used to clarify the associated molecular mechanisms.

**Results**: Circ0036602 was upregulated in HPV-16 positive CC and correlated with a poor prognosis. Moreover, circ0036602 expression significantly correlated with the clinicopathologic characteristics. Knockdown of circ0036602 inhibited CC cell proliferation, migration, and invasion. Further studies showed that circ0036602 could bind to miR-34-5p and miR-431-5p to regulate the expression of the target gene HMGB1.

**Conclusions**: Taken together, our findings suggest that circ0036602 is a tumor-promoting circRNA that promotes CC cells by sponging miR-34-5p and miR-431-5p to regulate HMGB1. Circ0036602 has huge prospects as a potential therapeutic target for CC patients.

## Introduction

Cervical cancer (CC) is the most common gynecological cancer and has become the second leading cause of cancer-related deaths among women 1, 2. There are about 570,000 new cases reported and 311,000 deaths annually 1. Although HPV vaccines have shown promising results over the past decades, they are prophylactic and have no benefit on active disease infection 3, 4. In addition, multivalent vaccines do not cover all types of HPV known to cause CC 5. It has been reported that more than 50% of cervical cancer patients are infected with HPV-16, documented as the predominant type in CC 3, 6. Interestingly, recent studies have demonstrated that the carcinogenicity of HPV-16 is associated with the highly conserved E7 protein that can promote the occurrence and development of HPV-16 positive cervical cancer 7. Accordingly, it is of great significance to investigate the molecular mechanisms, and explore prognostic and therapeutic targets for CC.

CircularRNAs (CircRNAs) are a class of single-stranded RNA characterized by a covalently closed loop short of the 5′ caps and 3′ poly-A tails, making them more stable than linear RNAs 8-10. They are endogenous, conserved, and abundant 11. CircRNAs have long been regarded as splicing byproducts with little functional potential 12. In recent years, emerging evidence has revealed that circRNA can exert functions as microRNAs (miRNAs) sponge 13, competing for miRNA binding, or gene transcription modulators to regulate target genes expression 14 and interact with RNA-binding proteins 15, 16. Moreover, many circRNAs have been demonstrated to be involved in various cancer-related biological processes, including cell metastasis, apoptosis and proliferation in CC 17, 18. For example, circFNDC3B inhibits colorectal cancer metastasis, invasion, and angiogenesis by sponging miR-937-5p to derepress TIMP3 expression 19. Moreover, circRNA NFATC3 may act as a miR-9-5p sponge and regulate the SDC2/NF-kB signaling pathway in CC 20. Besides, circ0067934 upregulation in CC reportedly promotes disease progression via the miR-545/EIF3C axis 21. These studies indicated that circRNA could be used as candidate biomarkers and therapeutic targets. With the development of high-throughput sequencing and advanced bioinformatics, an increasing number of circRNAs has been discovered in mammalian cell lines and across various species; however, their regulatory roles and clinicopathological relevance in CC are not well understood and need further exploration.

This study found that circ0036602 was upregulated in HPV-16 positive CC, deprived of exons (exons 5~10) of the ZNF592 gene and negatively associated with differentiation, N stage, and poor prognosis. Furthermore, our data demonstrated that circ0036602 silencing inhibited CC cell proliferation and invasion by acting as a sponge of miR-34a-5p and miR-431-5p. Overall, our findings suggest that circ0036602 is an oncogene and has huge prospects as a novel biomarker and a therapeutic target for CC.

## Methods

### Human species

In total, 30 HPV-16 positive CC tissues and matched HPV-16 negative CC tissues were harvested at the Jiangsu cancer hospital from June 2018 to July 2020 and stored in liquid nitrogen for later use. None of these patients received chemotherapy and radiotherapy prior to specimen collection. Written informed consent was obtained from all participants, and the study was approved by the Ethics Committee of The Affiliated Cancer Hospital of Nanjing Medical University. The study protocol was performed in accordance with the ethical standards of the Declaration of Helsinki. All collected tissues were examined by experienced pathologists.

### Cell Culture

CC cell lines Caski, Siha, C33A and normal cervical epithelial Hacat, purchased from American Type Culture Collection (ATCC), were cultured in Roswell Park Memorial Institute (RPMI) 1640 medium with 10% fetal bovine serum (FBS) and 1% penicillin at 37°C with 5% carbon dioxide.

### Cell Transfection

Small interfering RNAs (siRNA) targeting circ0036602, and siRNA control were designed by Ribobio (Guangzhou, China). Caski and Siha cells were cultured in a 96-well plate, incubated overnight at 37°C, and transfected when the cells were grown to 50~60% confluence. After 48h transfection, the cells were harvested, and the transfection efficiency was assessed by quantitative real-time PCR (qRT-PCR) analysis. Lipofectamine 2000 was used for transient transfection according to the manufacturer's protocol.

### Genomic DNA, RNA extraction, and quantitative real-time PCR analysis

Genomic DNA (gDNA) was extracted from cultured cells using a Blood & Cell Culture DNA Mini Kit (Qiagen, CA, USA), as described in the manufacturer's instructions. Total RNA was isolated from CC tissues or cell lines with Trizol reagent (Invitrogen, Life Technologies, Inc, Germany) following the standard protocol. The purity and concentration were detected with a Nanodrop2000 spectrophotometer. Complementary DNA (cDNA) was synthesized with 1 ug of total RNA using PrimeScript RT Reagent kit (Takara, Japan). Quantitative real-time PCR analysis was carried out with SYBR Green Real-time PCR Master Mix (Takara, Japan). The PCR procedure consisted of predegeneration at 95 ℃ for 20 s, followed by 40 cycles of degeneration at 95 ℃ for 10 s, and extension at 60 ℃ for 45 s. All data were presented as mean ± standard deviation of three independent experiments, and the relative gene expression levels were analyzed using the 2-ΔΔCq method normalized to endogenous control genes U6 and GAPDH. The primers utilized are as follows: ZNF592 sequence: (5'->3') Forward Primer: GAATGAGAGTCCCCTCAAACCT Reverse Primer TGTAGGAGACTGGGAGTAATGTG; Circ-0036602 (5'->3'): Forward Primer: CTTGTGTGCATGAATCCGCT, Reverse Primer: CTTGTGTGCATGAATCCGCT; miR-34a-5p: Forward Primer: GCAGTGGCAGTGTCTTAG, Reverse Primer: GGTCCAGTTTTTTTTTTTTTTTACAAC; miR-431-5p: Forward Primer: TCTTGCAGGCCGTCA; Reverse Primer: GTCCAGTTTTTTTTTTTTTTTGCAT; U6: Forward Primer: 5′-CTCGCTTCGGCAGCACA-3′, Reverse Primer: 5′-AACGCTTCACGAATTTGCGT-3′; GAPDH: Forward Primer: 5′-AAGAAGGTGGTGAAGCAGGC-3′, Reverse Primer: 5′-GTCAAAGGTGGAGGAGTGGG-3′.

### RNA isolation of nuclear and cytoplasmic fractions

The Nuclear and cytoplasmic RNA Purification Kit (Norgen, USA) was used to extract nuclear and cytoplasmic fractions based on the manufacturer's instructions. Then, total RNA was extracted using TRIzol reagent (Invitrogen, USA) for qRT-PCR as described above.

### RNA fluorescent *in situ* hybridization (FISH)

A specific probe targeting circ0036602 was designed and purchased from Genesee Biotech (Guangzhou, China). The cell nucleus was stained with 4,6-diamidino-2-phenylindole (DAPI) for 20 min and then imaged under a fluorescence microscope. The FISH experiment was performed using a fluorescence *in situ* hybridization kit (Ribobio, Guangzhou) to visualize the location of circ0036602 in the cell.

### RNase R Digestion

RNase R is a 3′ to 5'exoribonuclease that can digest circ0036602, and its linear counterpart ZNF592 mRNA. 2 μg RNAs were incubated with or without RNase R (Epicentre Technologies, Madison, USA) at 37°C for 30 min. Then, the relative expression levels of circ0036603 and ZNF592 mRNA were detected by qRT-PCR.

### Luciferase Reporter Assay

We obtained the potential binding sites of microRNA and circ0036602 via TargetScan and Miranda, and the sequences were cloned into a psiCHECK2 vector (Promega Corporation, USA). Cell lines were seeded in 48-well plates and co-transfected with the luciferase reporter vector, and microRNA mimic or negative control with lipofectamine 2000. The relative luciferase activity was measured after 2 days of transfection by a dual-luciferase assay system (Promega Corporation, USA), according to the manufacturer's instructions.

### RNA immunoprecipitation (RIP) assay

The putative relationship between circ0036602 and miR-34a-5p, miR-431-5p was further investigated by the RNA Immunoprecipitation (RIP) assay. The RIP assay was carried out using the EZ-Magna RIP kit (Millipore, USA). Briefly, cells were co-transfected with miR-34a-5p mimics or miR-431-5p mimics. The cells were harvested after 48h and lysed in RIP lysis buffer. Then, the cells were incubated with proteins magnetic beads conjugated with anti-ago2 or anti-IgG antibody (Millipore, USA) at 4 °C overnight. After washing with buffer, immunoprecipitated RNA was extracted. The abundance of circ0036602 and microRNA were measured by qRT-PCR.

### Cell Proliferation Assay

The Cell Counting Kit-8 was applied to determine cell viability at different time points (0h, 24h, 48h, 72h, 96h, 120h), according to the manufacturer's protocol. The Caski cells in the logarithmic phase were seeded into a 96-well plate containing a volume of 100μl per well. The cells transfected with siRNA1 and siRNA2 were seeded into six replicates. 10μl CCK-8 (Solarbio, China) was added to each well and incubated for 1h at 37 °C. Finally, the absorbance was measured at 450 nm wavelength using a micro-plate, and the cell viability was calculated. All experiments were performed in triplicate.

### Wound healing assay

Caski cells were divided into three groups: negative control (NC), siRNA-1, and siRNA-2 groups based on the treatment conditions. For the wound healing assay, transfected cells were seeded into 6-well plates and incubated at 37°C overnight until 100% confluence. A 10μl pipette tip was used to create a linear scratch and washed the detached cells with PBS. The distance of each scratch closure was subsequently observed and photographed via microscopy (100× magnification) at 0h and 48h. Every assay was performed in triplicate.

### Transwell assay

The Transwell assay was applied to assess the potential of cell migration and invasion. Transfected cells were cultured into the upper chamber containing 100μl serum-free medium, and the lower chamber contained 600μl complete medium harboring 10% FBS as a chemoattractant. After the cells were cultured at 37 °C for 24h, a cotton swab was used to gently remove cells from the upper cavity. Subsequently, the migrated cells were fixed with 4% paraformaldehyde for 20 min and dyed with 0.2% crystal violet. Finally, six random fields were observed under a microscope and photographed to measure the migrated cell count. The cell invasion assay was simultaneously performed with the above steps, except that the upper transwell chamber was pre-coated with matrigel (corning, USA).

### Western blot

Total protein was extracted from CC tissues and cells using the RIPA (radioimmunoprecipitation assay) lysis buffer (Beyotime, China), and the protein concentration was measured by the BCA protein assay kit (Beyotime, China). 25μg protein samples were separated by SDS-PAGE gels, and transferred to PVDF membrane (Thermo Fisher Scientific). After blockage with 5% skimmed milk at room temperature for 2h, the PVDF was incubated with the primary antibody against E7 (1:800, Invitrogen, USA), ZNF592 (1:500, Abcam, USA), HMGB1 (1:1000, Abcam, USA), or GAPDH (1:2000, Santa Cruz biotechnology, USA) at 4°C overnight. Then, the membranes were washed by PBS three times and incubated with the secondary antibody (anti-rabbit, 1:10000, Thermo Fisher Scientific, USA) for 1h at room temperature. After washing 3 times using PBST, the membranes were photographed by the ECL detection system. ImageJ software was used to analyze the western blot bands.

### Statistical analysis

All analyses were performed using SPSS 20.0 statistical software. Comparison between two experimental groups was performed by the Student's t-test. ANOVA was used for comparisons of more than two groups. The Kaplan-Meier analysis was used to plot survival curves, and the log-rank test was used to compare the survival curves. Data were presented as the mean ± SD. P values < 0.05 were statistically significant.

## Results

### Circ0036602 was upregulated in CC

**Figure 1:**
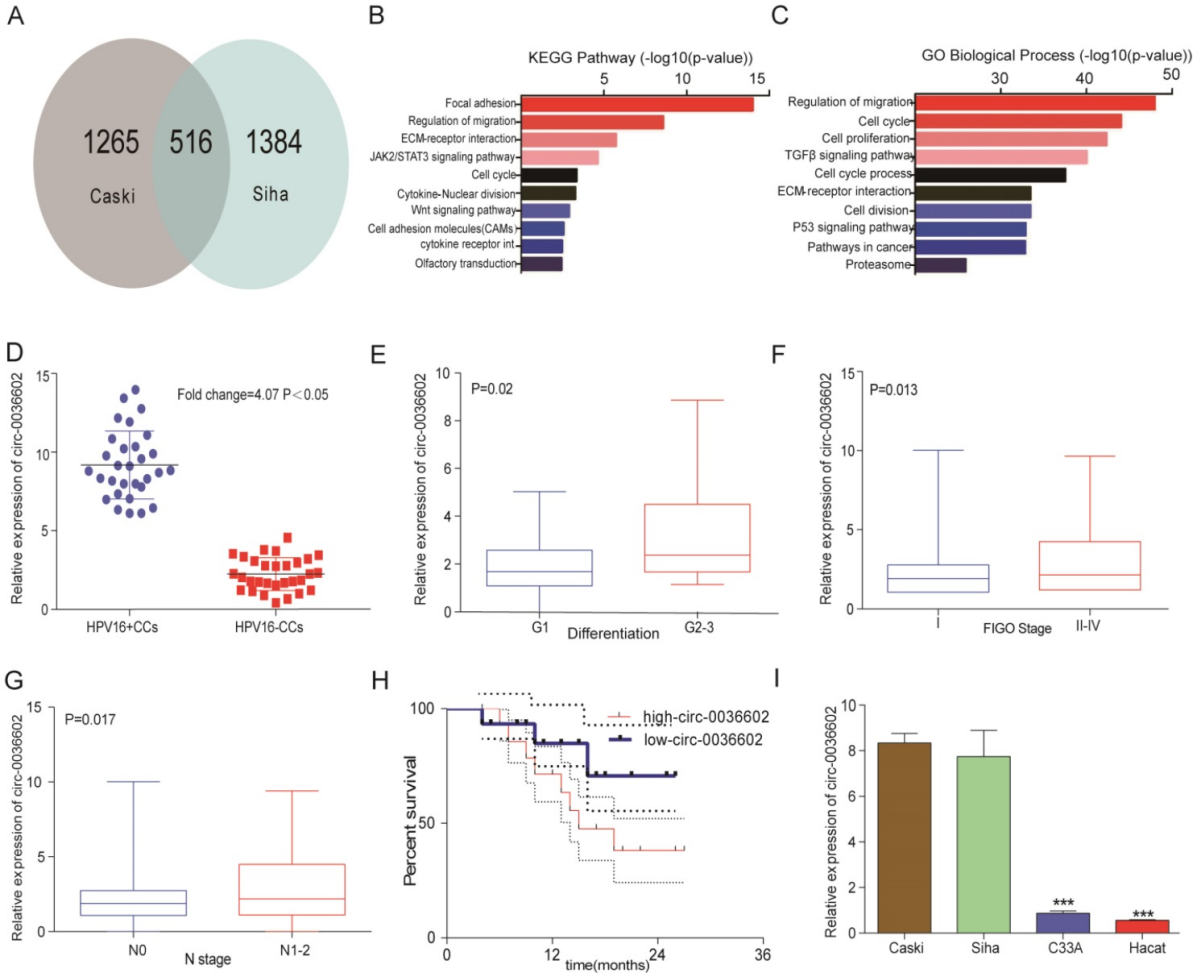
Circ0036602 was upregulated in CC. (A) A total of 516 differentially expressed cirRNAs in Caski-shRNA-E7, Siha-shRNA-E7, and negative control were identified via cirRNAs RNA sequencing. (B-C) Kyoto Encyclopedia of Gene and Genome pathway and Gene Ontology analysis of differentially expressed genes in cervical cancer. (D) The relative expression of circ0036602 in HPV16 positive and HPV negative CC tissue samples. (E-G) The correlated analysis between the relative expression of circ0036602 and differentiation, FIGO stage, and N stage. (H) Kaplan-Meier curves of overall survival of 30 CC patients with low or high circ0036602 expression. (I) The relative expression of circ0036602 in CC cells performed by qRT-PCR. *p< 0.05, Values represent mean± SD, n= 3 independent experiments.

To identify novel circRNAs involved in the progression of HPV positive CC, cervical cancer cell lines Caski and Siha were selected to construct stable transgenic cell lines with E7 oncoprotein knockdown (Caski-shRNA-E7, Siha-shRNA-E7). We performed a circRNA expression profiling microarray analysis on the transgenic (Caski-shRNA-E7, Siha-shRNA-E7) and control cell lines (Caski-NC, Siha-NC). 516 circRNAs were found in both microarrays (Figure 1A). The differentially expressed circRNAs were identified based on the selection criteria: | fold change (FC) | ≥ 4 and p-values < 0.05. Kyoto Encyclopedia of Genes and Genomes (KEGG) analysis revealed that differentially expressed circRNAs were significantly enriched in focal adhesion, regulation of migration, ECM-receptor interaction and JAK2/STAT3 signaling pathway (Figure 1B). Gene ontology (GO) analysis of differentially expressed circRNAs showed that regulation of migration, cell cycle, cell proliferation and TGF-β signaling pathway were significantly enriched (Figure 1C). The top 3 upregulated and downregulated circRNAs were selected for further analysis (Table 1), and primers for these circRNAs were synthesized. The expression of these circRNAs was measured in 30 paired HPV positive and HPV negative tissue samples. Among them, circ0036602 was the most significantly upregulated differentially expressed circRNA by approximately 4.07-fold (Figure 1D, Figure S1A). Therefore, circ0036602 was chosen for further analysis. Subsequently, we analyzed the correlation between the expression of circ0036602 and clinicopathological features. The results indicated that circ0036602 expression was positively correlated with degree of differentiation (p = 0.02, Figure 1E), FIGO stage (p = 0.013, Figure 1F) and N stage (p = 0.017, Figure 1G). Moreover, high circ0036602 expression resulted in shorter patient's survival (p = 0.01, Figure 1H). In addition, four cervical cancer cell lines and a normal cervical epithelial cell line were used to assess the expression of circ0036602. In line with the results of circ0036602 expression in cancer tissue, circ0036602 was significantly upregulated in HPV-16 positive cells (Caski and Siha) compared to HPV-16 negative (C33A) and normal cells (Hacat) (Figure 1I). Taken together, these data implied that circ0036602 was upregulated and might be involved in CC progression.

### The characterization of circ0036602 in CC

**Figure 2:**
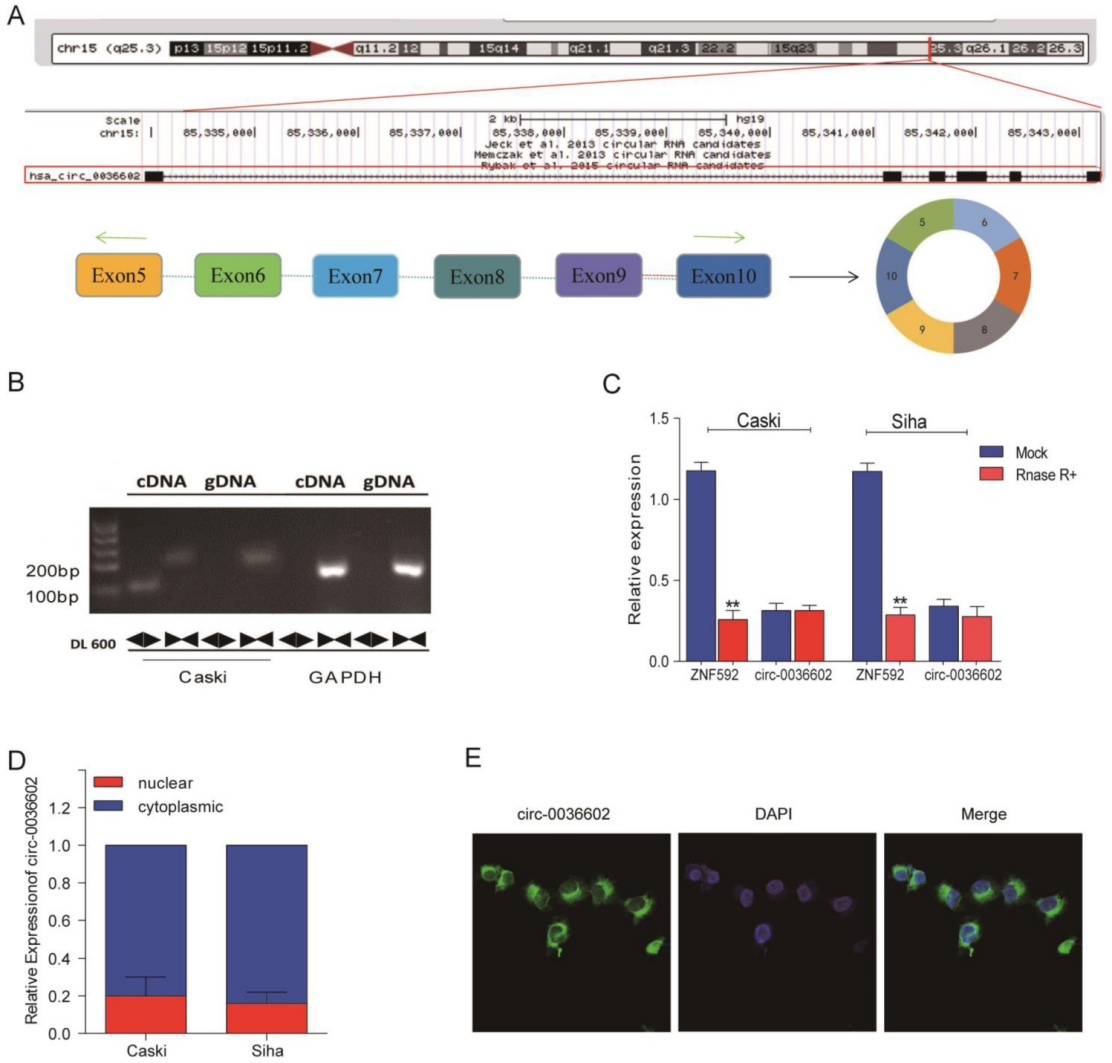
The characterization of circ0036602 in CC. (A) The schematic illustration showed that exons 5~10 of ZNF592 constitute circ0036602. (B) The presence and circular form of circ0036602 established by agarose gel electrophoresis. GAPDH acts as the linear control. (C) circ0036602 and ZNF592 mRNA expression in Caski and Siha cells treated with or without R detected by qRT-PCR analysis. (D-E) The nuclear and cytoplasmic distribution of circ0036602 assessed by qRT-PCR and FISH assay in. *p< 0.05, Values represent mean± SD, n= 3 independent experiments.

To further characterize circ0036602, data from the CircBase online database was analyzed. As demonstrated in Figure 2A, circ0036602 was located on chromosome 15q23.3 and consisted of six exons, spanning from exons 5 to 10 in the host ZNF592. Moreover, we designed a divergent primer and found that circ0036602 could only be amplified in complementary DNA (cDNA) rather than in genomic DNA (gDNA) (Figure 2B). Then, we found that circ0036602 was resistant to digestion by Rnase R, while linear ZNF592 was almost completely degraded (Figure 2C), confirming the circular structure of circ0036602. Additionally, to explore the subcellular location of circ0036602, the nuclear fraction was isolated from the cytoplasmic. qRT-PCR showed that circ0036602 was mainly distributed in the cell cytoplasm (Figure 2D). Moreover, FISH staining confirmed that circ0036602 was predominantly located in the cytoplasm (Figure 2E).

### Silencing circ0036602 suppressed CC invasion, migration and proliferation

**Figure 3:**
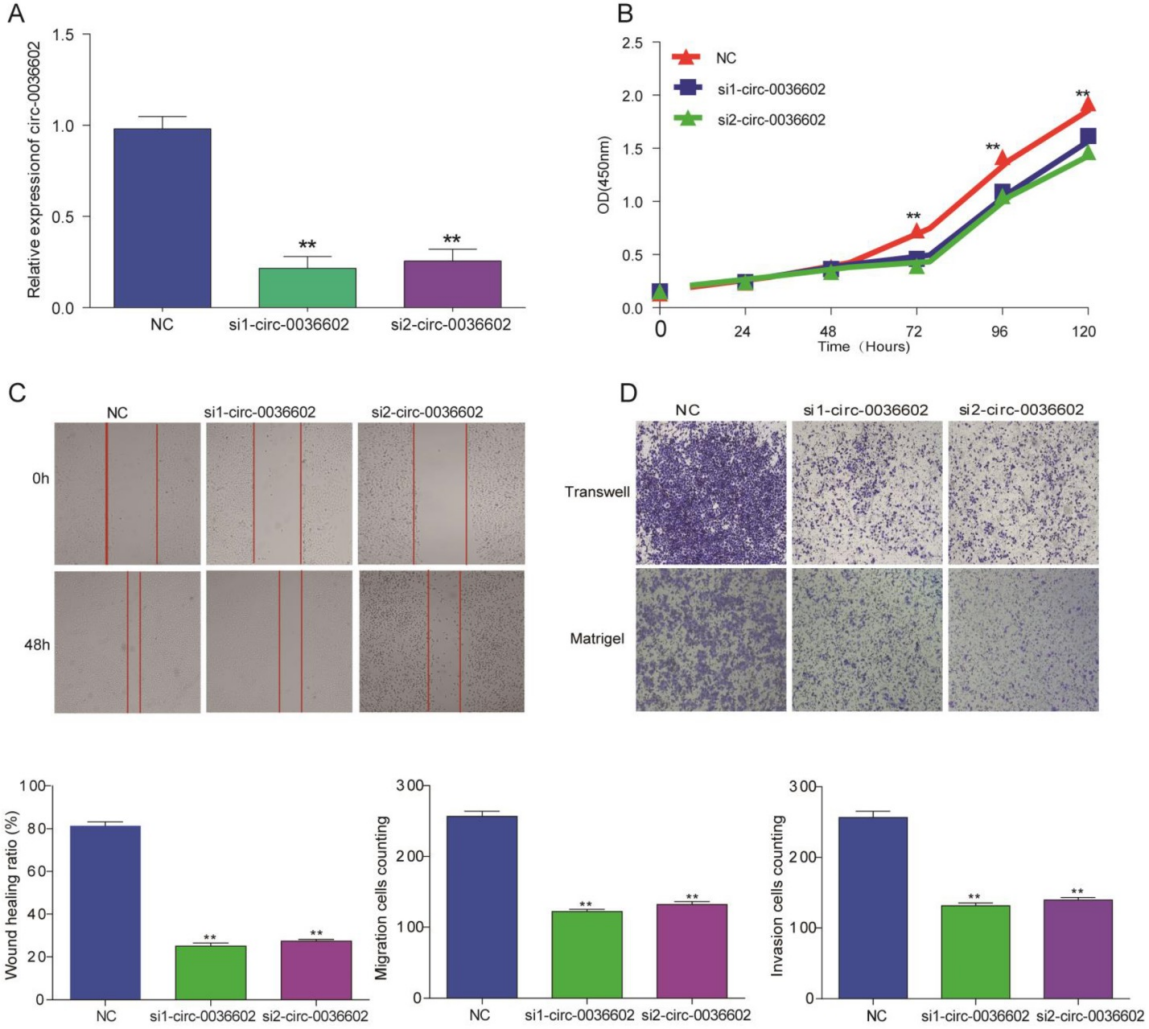
Silencing circ0036602 suppressed CC invasion, migration, and proliferation. (A) The relative expression of circ0036602 in Caski cells transfected with si1-circ0036602, si2-circ0036602, or siNC examined by qRT-PCR. (B) CCK-8 assays were performed to examine the cell proliferation rate at 0, 24, 48, 72 and 96h. (C) Wound healing assays showed decreased cell migration ability of Caski cells. (D) Transwell migration and invasion assay confirmed knockdown circ0036602 inhibited the migration and invasion ability. *p< 0.05, Values represent mean± SD, n= 3 independent experiments.

To explore the functional role of circ0036602 in CC cells, we designed siRNAs (siRNA1, siRNA2) specifically targeting circ0036602 and transfected into Caski cells to silence circ0036602. qRT-PCR results demonstrated that both siRNAs could decrease circ0036602 expression, as shown in Figure 3A. Subsequently, we performed CCK8 assays to investigate the effect of silencing circ0036602 on CC cell proliferation. As shown in Figure 3B, the proliferation ability of Caski cells was found to be impeded after circ0036602 silencing. Wound healing assays demonstrated that knockdown of circ0036602 significantly restrained cell migration in Caski (Figure 3C). Additionally, to further clarify whether circ0036602 affects cell migration and invasion, transwell migration and invasion assays also confirmed that the migration and invasion ability of Caski cells were inhibited after circ0036602 depletion (Figure 3D). Together, these results indicated that silencing circ0036602 inhibited migration, invasion, and proliferation of CC cells.

### Circ0036602 acted as a sponge of miR-34a-5p and miR-431-5p in CC

**Figure 4:**
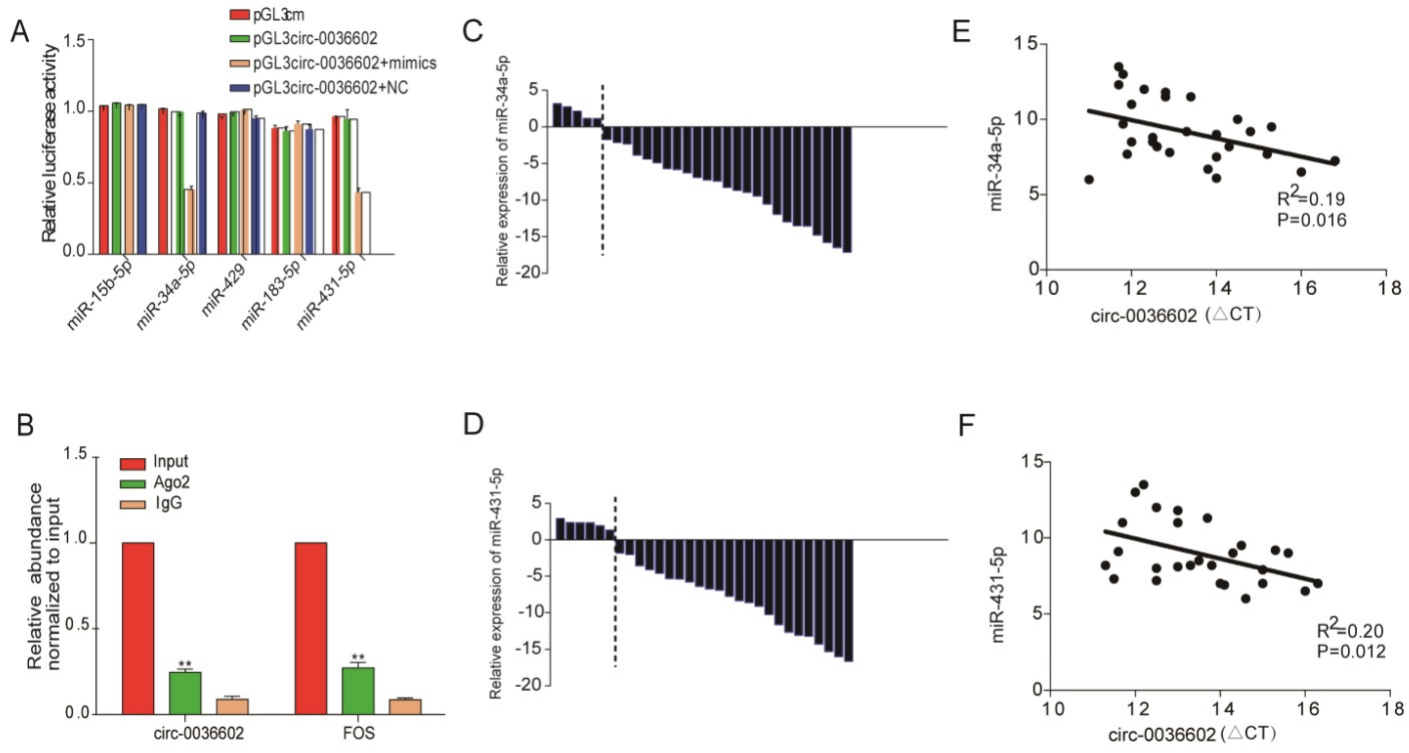
Circ0036602 acted as a sponge of miR-34a-5p and miR-431-5p in CC. (A) Luciferase reporter gene assays confirmed that miR-34a-5p and miR-431-5p can bind with circ0036602. (B) Ago2 RIP experiment showed the combination of Ago2 protein with circ0036602. (C-D) Relative experssion of miR-34a-5p and miR-431-5p in CC and control tissues by qRT-PCR. (E-F) Correlation between circ0036602 and miR-34a-5p and miR-431-5p in CC tissues. *p< 0.05, Values represent mean± SD, n= 3 independent experiments.

Numerous studies have confirmed that circRNAs, which are mainly localized in the cytoplasm, can interact with miRNAs to regulate the expression of downstream target genes 22, 23. We thus searched for potential miRNAs associated with circ0036602 using the TargetScan, PITA, and Miranda databases. In total, 5 miRNAs were identified as putative targets, including miR-15b-5p, miR-34a-5p, miR-429, miR-183-5p and miR-431-5p. To further assess whether circ0036602 could bind to these miRNAs, we performed dual-luciferase assays (Figure 4A). CC cells transfected with miR-34a-5p or miR-431-5p mimics significantly exhibited decreased luciferase activities than the negative control. It has been established that miRNAs can combine with Ago2 to form an RNA-induced silencing complex (RISC) to silence gene expression. Accordingly, the RNA immunoprecipitation (RIP) assay showed that anti-Ago2 suppressed circ0036602 expression (Figure 4B). Therefore, we speculated that circ0036602 might function through competitive binding to miR-34a-5p and miR-431-5p. Previous studies have shown that miR-34a-5p and miR-431-5p were potential oncogenes and were downregulated in various solid tumors, including CC, hepatocellular and colorectal cancer 24-26. To confirm the expression of both miRNAs in CC, qRT-PCR was performed, and the results indicated that both miRNAs were significantly downregulated in HPV-16 positive CC tissues compared with HPV-16 negative ones (Figure 4C and 4D). Moreover, the expression of circ0036602 was negatively correlated with miR-34a-5p and miR-431-5p in CC tissues (Figure 4E and 4F). The above results indicated that circ0036602 could serve as a sponge of miR-34a-5p and miR-431-5p to regulate their expression negatively.

### HMGB1 was a target of miR-34a-5p and miR-431-5p

**Figure 5:**
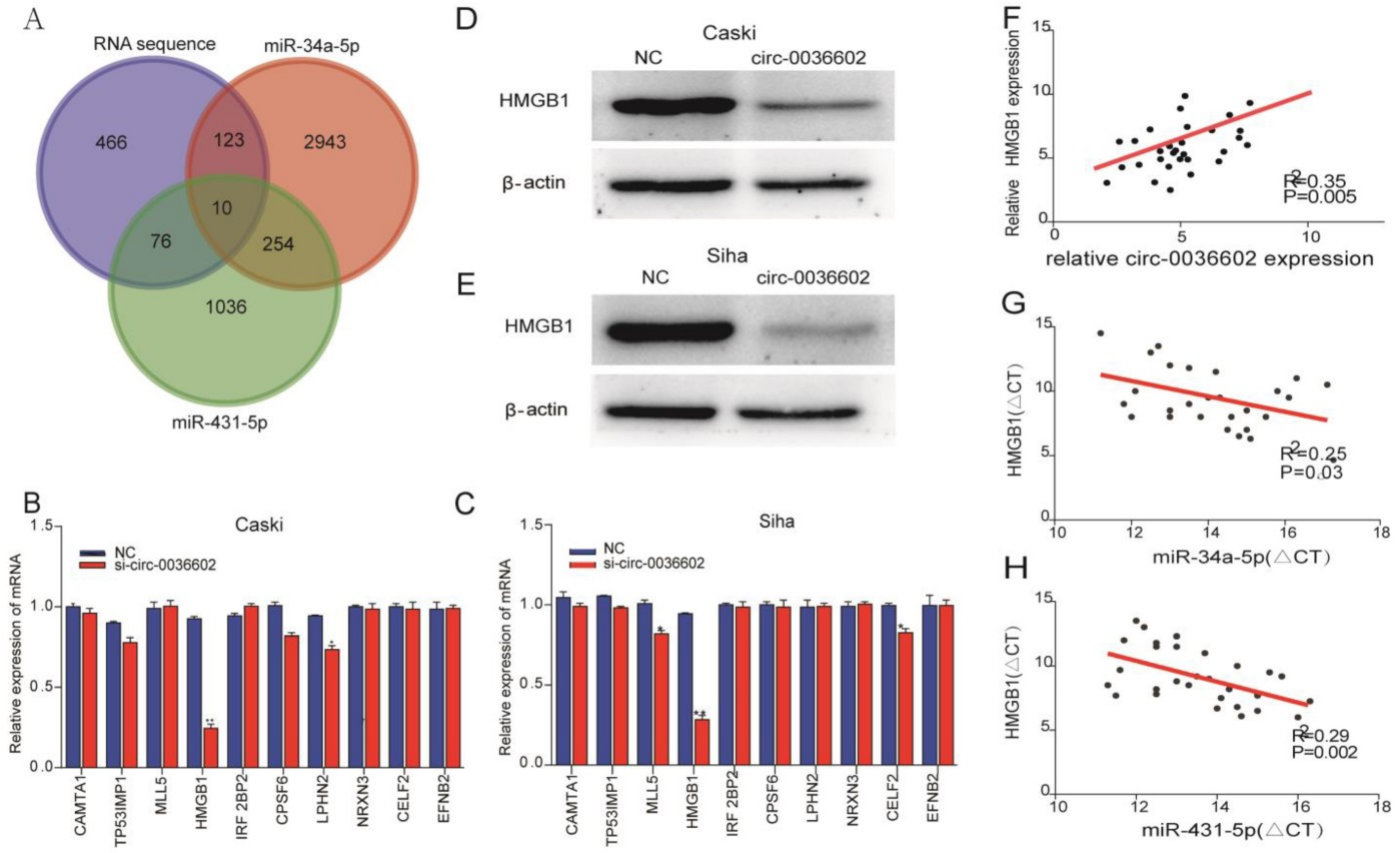
HMGB1 was a target of miR-34a-5p and miR-431-5p. (A) Venn diagram demonstrated the 10 genes from miRbase, TargetScan, Miranda, and RNA sequencing. (B-C) The relative expression of 10 genes in Caski and Siha cells detected by qRT-PCR. (D-E) Western blot verified the expression of HMGB1 in Caski and Siha cells. (F) Correlation analysis between circ0036602 and HMGB1. (G-H) The negative intersection with miR-34a-5p and miR-431-5p and HMGB1. *p< 0.05, Values represent mean± SD, n= 3 independent experiments.

Previous studies discovered that miRNA could regulate gene expression by binding to 3'-UTR of mRNA 27. To reveal the potential downstream target genes of both siRNAs mentioned above, CC Caski cells were selected and transfected with si-circ0036602 for RNA sequencing. Cluster analysis demonstrated that 1300 genes were upregulated while 460 were downregulated. Several online prediction tools, including miRase, TargetScan and Miranda were used to identify the miRNA targets. Results showed that 2943 candidates were screened from miR-34a-5p and 1036 from miR-431-5p. The intersection of the candidate genes yielded 10 genes, namely CAMTA1, TP531NP1, MLL5, HMGB1, IRF2BP2, CPSF6, LPHN2, NRXN3, CELF2, and EFNB2 (Figure 5A). Next, we quantified their expression by qRT-PCR and found that HMGB1 was significantly downregulated in Caski and Siha cells (Figure 5B and 5C). Consistently, western blot analysis showed that HMGB1 was downregulated following knockdown of circ0036602 (Figure 5D and 5E). Meanwhile, we found a significant positive correlation between HMGB1 and circ0036602 (Figure 5F) and a negative correlation between HMGB1 with miR-34a-5p (Figure 5G) and miR-431-5p (Figure 5H), which demonstrated that HMGB1 is a target of miR-34a-5p and miR-431-5p, and indirectly regulated by circ0036602.

## Discussion

Discovering novel diagnostic and prognostic biomarkers and unraveling the pathogenesis underlying HPV-16 positive CC is essential to refine current CC treatment efficacy. Numerous circRNAs have been identified in recent years, and their aberrant expression in various cancer have attracted significant attention. Furthermore, circRNAs have been documented as new diagnostic biomarkers and therapeutic targets for cancers 28, 29. Many circRNAs contain multiple miRNA binding sites and competitively bind with miRNA to mediate its activity 30. Notwithstanding that several circRNAs have been reported to participate in CC development 31, 32 , the role of circ0036602 in HPV16-positive CC remains unclear. To the best of our knowledge, this is the first study to investigate the association between circ0036602 and CC and explore the molecular mechanisms of migration and invasion in HPV-16 positive CC. In the current study, we performed RNA sequencing on E7-knockdown CC cells and identified six circRNAs. Finally, we found that a new circRNA generated from the ZNF592 gene was significantly elevated in HPV-16 positive CC tissues relative to HPV16 negative ones. Moreover, circ0036602 was upregulated in HPV-16 positive CC cells, and high circ0036602 expression was negatively correlated with the degree of differentiation, N stage, and patient survival, suggesting the involvement of circ0036602 in CC. Besides, functional assays indicated that circ0036602 could promote growth and stimulate migration and invasion.

Subsequently, the dual-luciferase reporter and RIP assays showed that miR-34a-5p and miR-431-5p could bind circ0036602 in CC cells. The function of miR-34a-5p or miR-431-5p has been documented in the literature. Wang et al. demonstrated that miR-34a-5p overexpression could inhibit cell viability, migration, invasion and promote apoptosis via Bcl-2 downregulation in cervical cancer 24. Moreover, Gao et al. showed that miR-34a-5p expression was lower in patients with colorectal cancer recurrence than without recurrence and could inhibit metastasis and recurrence in a p53-dependent manner 25. Sun et al. identified miR-431-5p as a tumor-suppressor miRNA that negatively regulated ZEB1. By targeting ZEB1, miR-431-5p could inhibit the epithelial-mesenchymal transition induced by ZEB1 26. Concordant with the literature, our study showed that miR-34a-5p was downregulated in CC samples and exerted antitumor effects. However, to the best of our knowledge, no studies have documented the regulatory role of miR-431-5p. To identify the target genes of both miRNAs, bioinformatics analysis and RNA sequencing were used, and HMGB1 was identified. Western Blot and qRT-PCR revealed that HMGB1 was upregulated and positively correlated with circ0036602, indicating that HMGB1 is a potential target of miR-34a-5p and miR-431-5p.

HMGB1 is one of the highly conserved chromatin-associated proteins containing HMG-box domains and has been reported to be abnormally expressed in diverse cancers 33. Chen et al. reported that HMGB1 played a novel role in regulating the Hippo pathway and promoted liver tumorigenesis in an HMGB1-YAP dependent pathway 34. Furthermore, Gao et al. found that HMGB1 acted as the downstream gene of lncRNAZEB2/miR-204/HMGB1 to promote pancreatic cancer cell growth and invasion 34. Moreover, Li et al. indicated that HMGB1 was upregulated and can be an independent predictor and prognostic biomarker in cervical cancer, consistent with our study findings 35. In the present study, RNA sequencing and online database analysis showed that HMGB1 is a potential target of miR-34a-5p and miR-431-5p. Moreover, we observed that HMGB1 expression was inversely correlated with both miRNAs, and positively correlated with circ0036602, supporting the hypothesis that HMGB1 is its downstream target. However, further studies are required to substantiate the regulatory role of circ0036602 in CC. In addition, it is conceivable that other circRNAs are involved in the progression of CC, warranting the need for further studies.

## Conclusion

Taken together, this is the first study to document the oncogenic function of circ0036602 in CC. In this regard, circ0036602 blocked the migration, invasion, and proliferation of CC by HMGB1 upregulation mediated by miR-34a-5p and miR-431-5p. These findings revealed that circ0036602 has huge prospects as a therapeutic target and diagnostic biomarker for CC.

## Supplementary Material

Supplementary figure.

## Figures and Tables

**Table 1:** 3 up-regulated and 3 down-regulated circRNAs were picked up by circRNA expression profiling microarray analysis.

cirRNA	chromosome	circRNA-type	Gene symbol	Spliced length (bp)	regulation
Circ-0011968	chr5	exonic	MTF2	1064	up-regulation
circ-0005092	chr14	exonic	UBE2E2	341	up-regulation
circ-0036602	chr15	exonic	ZNF592	1053	up-regulation
circ-0013306	chr6	exonic	CNOT4	1719	down-regulation
circ-0008995	chr1	exonic	MFF	588	down-regulation
circ-0008731	chr1	exonic	MAP4K4	1375	down-regulation

## Abbreviations

CC: Cervical Cancer
qRT-PCR: quantitative real-time PCR
CCK8: Cell Counting Kit-8 assay
GO: gene ontology
KEGG: Kyoto Encyclopedia of Genes and Genomes
ATCC: American Type Culture Collection
RPMI: Roswell Park Memorial Institute
FBS: fetal bovine serum
RIP: RNA Binding Protein Immunoprecipitation
