# Supplementary figures and images for: Circ-0036602 Acts As a Sponge of MiR-34a-5p and MiR-431-5p to Promote Cervical Cancer Proliferation and Invasion
Source: J Genomics. 2022 Jan 11;10:16–25. doi: 10.7150/jgen.62458 (PMC8824728; doi:10.7150/jgen.62458)

Figure S1.

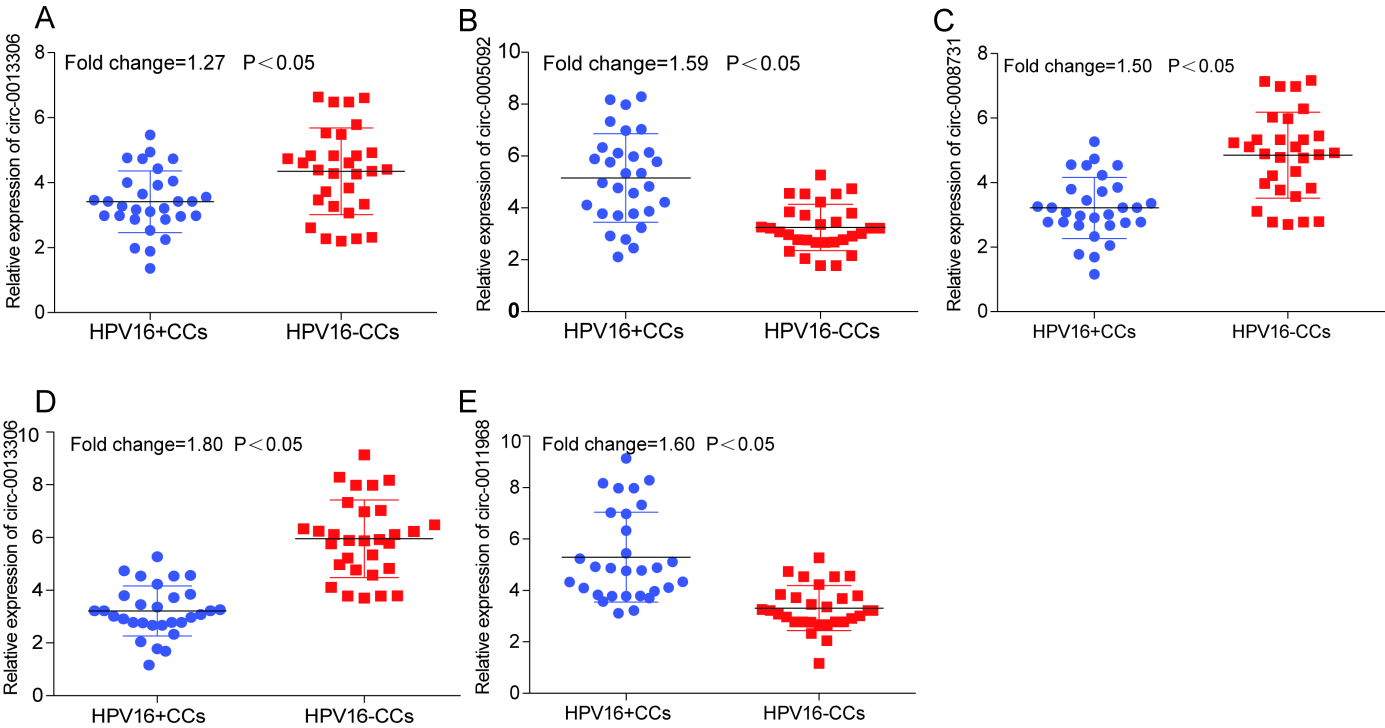

Supplement: Supplementary file 1 — Supplementary figure. [file jgenv10p0016s1.pdf]
